# GPR50 in TGFβ signaling and breast cancer

**DOI:** 10.18632/oncoscience.433

**Published:** 2018-06-27

**Authors:** Raise Ahmad, Stefanie Wojciech, Ralf Jockers

**Affiliations:** Inserm, U1016, Institut Cochin, Paris, France; CNRS UMR 8104, Paris, France; Univ. Paris Descartes, Sorbonne Paris Cité, Paris, France

**Keywords:** GPR50, orphan GPCRs, TGFβ, breast cancer, smad signaling

G protein-coupled receptors (GPCRs) are involved in many physiological and pathophysiological processes including cancer development. However, many functions of these receptors remain unknown, especially for the orphan GPCRs that do not have an identified ligand. We demonstrate here, for the first time, the anti-tumor properties of an orphan GPCR, GRP50. In mammary tumors, the formation of heterodimers between GRP50 and the type I TGFβ receptor (TβRI) activates the antiproliferative effect of the TGFβ receptor which results in a lower tumor growth. Moreover, in women with breast cancer, low expression of GPR50 has been associated with poor survival prognosis regardless of the subtype of breast cancer. These new results make GRP50 a promising diagnostic tool and therapeutic target for breast cancer [1].

GPCRs, also called 7 transmembrane domain receptors, represent the largest family of cell surface proteins with approximately 800 members in humans. They are involved in many physiological and pathophysiological processes and are targeted by nearly 30% of currently prescribed drugs. Two types of ligands/factors are known to modulate the function of GPCRs: orthosteric ligands and allosteric ligands or interacting partners. GPCRs recognize a wide variety of orthosteric ligands of either external (odors, light, molecules of taste etc.) or internal nature (hormones, neurotransmitters etc.). Approximately 100 GPCRs are still orphan for which no endogenous orthosteric ligand has been identified so far [2]. Knowing the function of orphan GPCRs is of high interest since they represent promising future drug targets. In this respect, we proposed the concept of ligand-independent functions of orphan GPCRs that relies on their capacity to allosterically regulate the function of other receptors in common protein complexes [3, 4]. In line with this idea we recently discovered a molecular complex between an orphan GPCR, GPR50 [5], and TGFβ receptor the type I (TβRI), which is a well-known player in cancer pathophysiology [6]. The association of GRP50 with TβRI leads to the spontaneous, ligand-independent, phosphorylation and activation of TβRI followed by the activation of the canonical smad2/3 and the non-canonical p38/MAPK downstream signaling pathways. Interestingly this effect requires neither the presence of the TGFβ receptor type II (TβRII) nor the TGFβ ligand. Mechanistically, GPR50 is replacing TβRII and activating downstream signaling in a constitutive manner. More precisely, GPR50 activates TβRI by promoting TβRI autophosphorylation and by preventing the binding of the negative regulator FKBP12 to TβRI. The latter effect is due to the competition between GPR50 and FKBP12 for TβRI binding through a common ATxzHP (x=Ala, Thr, Ser; z=Gly, Ser) motif. In terms of functional relevance, GPR50 behaves like TβRI by showing the anti-proliferative effect following overexpression and decreases tumor growth in a xenografted mouse model. Conversely, the targeted deletion of GPR50 in MMTV/Neu spontaneous mammary cancer increases tumor growth and decreases the chances of survival of the animal. In terms of the expression pattern of GPR50 in human breast cancer subjects it has been found that low expression of GPR50 is associated with a poor survival prognosis regardless of the cancer subtype. This observation is compelling enough to check GPR50 expression during breast cancer progression as it can provide a new tool for better prognosis and follow up of the disease. The potential relevance of the GPR50-TβRI complex in other tissues and cancer types is currently unknown but warrants further attention knowing the widespread expression pattern of TβRI. TGFβ signaling is known for its dual functionality in mammary tumor development as it appears inhibitory at early stage of tumorigenesis, whereas tumor cells at advanced stages can evade antiproliferative control and undergo tumorigenic progression in response to TGFβ [6].

Moreover, breast cancer starts as a local disease and can metastasize to distant organs. The conversion of early stage tumors into invasive malignancies has been associated with the activation of EMT, defined as changes in cell phenotype from an epithelial to a mesenchymal state, which are both a fundamental event and a hallmark in tumorigenesis. Presently there is no clear idea about the existence and functional ‘behaviour’ of the GPR50-TβRI complex in later stages of tumorigenesis or metastasis. It would be interesting to check whether GPR50 mimics the dual nature of TGFβ during the different stages of breast cancer or whether GPR50 remains protective all the way. Integrating the GPR50-TβRI complex into the equation will hopefully allow a better understanding of TGFβ biology and its paradox in breast cancer pathophysiology.
